# Efficacy and safety profile of *nab*-paclitaxel plus gemcitabine in patients with metastatic pancreatic cancer treated to disease progression: a subanalysis from a phase 3 trial (MPACT)

**DOI:** 10.1186/s12885-016-2798-8

**Published:** 2016-10-21

**Authors:** Arndt Vogel, Josefine Römmler-Zehrer, Jack Shiansong Li, Desmond McGovern, Alfredo Romano, Michael Stahl

**Affiliations:** 1 Department of Gastroenterology, Hepatology and Endocrinology, Medizinische Hochschule Hannover, Hannover, Germany; 2 Medizinische Hochschule Hannover, Ltd. Oberarzt der Klinik für Gastroenterologie, Hepatologie & Endokrinologie, Gebäude I11, Ebene H0, Raum 1380, Carl-Neubergstr. 1, 30625 Hannover, Germany; 3 Celgene Corporation, Summit, NJ USA; 4 Department of Medical Oncology, Kliniken Essen-Mitte, Essen, Germany

**Keywords:** Gemcitabine, Metastatic pancreatic cancer, *nab*-paclitaxel, Progressive disease, Subgroup analysis

## Abstract

**Background:**

The phase 3 MPACT trial in patients with metastatic pancreatic cancer demonstrated superior efficacy of *nab*-paclitaxel (*nab*-P) + gemcitabine (Gem) vs Gem monotherapy for all endpoints examined including overall survival, the primary endpoint. In the MPACT trial, patients were treated until progressive disease (PD) or unacceptable toxicity. The current exploratory analysis investigated outcomes of patients from the MPACT trial who were treated until PD, in order to understand how to maximize treatment benefit from *nab*-P + Gem.

**Methods:**

The trial design has been described in detail previously. Progressive disease was determined by the investigator on the basis of radiological imaging.

**Results:**

Among patients who were treated until PD, overall survival was significantly longer for those who received *nab*-P + Gem vs Gem (median, 9.8 vs 7.5 months; *P* < 0.001). Independently assessed progression-free survival and overall response rate were significantly greater among patients in the treatment-to-PD cohort who received *nab*-P + Gem compared with Gem (*P* < 0.001 for each). Although not compared statistically, patients who were treated until PD received greater treatment exposure and experienced more favourable efficacy than the intent-to-treat population of the MPACT trial. Among patients who were treated with *nab*-P + Gem until PD, > 50 % went on to receive a subsequent therapy. The safety profile for patients treated until PD was similar to what was reported in the overall MPACT trial.

**Conclusion:**

The *nab*-P + Gem regimen is an active first-line treatment option; most patients were treated until PD, and this exposure was associated with improved efficacy outcomes. Prolonged first-line treatment exposure and ability to receive subsequent therapies likely contributed to the improved survival among these patients. Our data highlight the importance of managing adverse events and indicate that patients should be treated until PD when possible.

**Trial registration:**

ClinicalTrials.gov NCT00844649 (MPACT trial); Registration date of this prospective phase III trial: February 13, 2009; current exploratory subanalysis was conducted retrospectively.

**Electronic supplementary material:**

The online version of this article (doi:10.1186/s12885-016-2798-8) contains supplementary material, which is available to authorized users.

## Background

Worldwide pancreatic cancer mortality and incidence rates are nearly equal [1]. In the United States and Europe, pancreatic cancer is the fourth leading cause of cancer-related mortality, with a 5-year survival rate of 7 % to 8 % among patients with all disease stages [2–4]. Surgical resection offers the only curative treatment for pancreatic cancer; however, only 15 % to 20 % of patients are candidates for surgery at diagnosis [5]. Even when an R0 resection is achieved, many patients will relapse within 2 years, and it is likely that distant micrometastases have already been established in the ≈ 15 % to 20 % of patients believed to be surgery candidates [6, 7]. According to the Surveillance, Epidemiology, and End Results Program, 52 % of patients with pancreatic cancer are diagnosed with metastatic disease, which portends a 2.6 % 5-year survival rate [4].

At the metastatic stage, the goals of treatment are to palliate symptoms and prolong survival [7]. Since the phase 3 trial nearly 20 years ago [8] that led to the approval of gemcitabine (Gem), numerous phase 3 trials of Gem combination regimens have failed to demonstrate a clinically and statistically significant survival benefit compared with Gem monotherapy in patients with metastatic pancreatic cancer [9–16]. Recently, 2 regimens, FOLFIRINOX (folinic acid + 5-fluorouracil [5-FU] + irinotecan + oxaliplatin) and *nab*-paclitaxel (*nab*-P) + Gem, demonstrated significantly longer survival compared with Gem alone [17–19]. The phase 3 MPACT trial (ClinicalTrials.gov NCT00844649) demonstrated superior efficacy of *nab*-P + Gem compared with Gem alone for all trial endpoints, including the primary endpoint, overall survival (OS; median, 8.7 vs 6.6 months; hazard ratio [HR], 0.72; *P* < 0.001) in patients with metastatic pancreatic cancer and Karnofsky performance status ≥ 70 [18, 19]. In the MPACT trial, grade ≥ 3 adverse events (AEs) were effectively managed by dose reductions and delays.

Although results from the phase 3 PRODIGE and MPACT trials were encouraging [17, 19], the regimens are not recommended for all patients with metastatic pancreatic cancer. A retrospective analysis found that 75 % of real-world patients with metastatic pancreatic cancer did not meet the PRODIGE trial inclusion criteria, with performance status, age, and elevated bilirubin levels being the main reasons for ineligibility [20]. The inclusion criteria of the MPACT trial [18] allowed for *nab*-P + Gem to be administered to a wider range of patients, including older patients or those with poorer performance status. Because *nab*-P + Gem has now become the most commonly used first-line chemotherapy option for patients with metastatic pancreatic cancer in the United States [21], it is important to better understand how to achieve the optimal benefit with this regimen. Per protocol, patients in MPACT were treated until progressive disease (PD) or unacceptable toxicity. The current exploratory analysis investigated characteristics and outcomes of patients who were treated until PD as assessed by radiological imaging.

## Methods

### Study design

Study design and patient eligibility of the phase 3 MPACT trial were described previously [18]. Patients were randomly assigned 1:1 to either intravenous *nab*-P 125 mg/m^2^ followed by intravenous Gem 1000 mg/m^2^ once weekly for the first 3 weeks of a 4-week cycle or Gem 1000 mg/m^2^ for the first 7 weeks of an 8-week cycle (cycle 1) and subsequently the first 3 weeks of a 4-week cycle (cycle ≥ 2). Per protocol, patients were treated until either PD or an unacceptable level of AEs. Tumour response was evaluated every 8 weeks using spiral computed tomography or magnetic resonance imaging. The aim of the present analysis was to determine the characteristics and outcomes of patients who were treated until PD during the phase 3 MPACT trial.

The PD cohort consisted of patients who experienced disease progression as declared by the investigator on the basis of computed tomography or magnetic resonance imaging and excluded patients who received further therapy. These patients also may have experienced a treatment-limiting toxicity at the time of PD. As a comparator group, patients who discontinued treatment due to AEs in the absence of PD were also analysed.

### Subsequent therapy use

Data on subsequent therapies included only the dates and type of treatment administered. For patients who received FOLFOX (folinic acid + 5-FU + oxaliplatin) or OFF (oxaliplatin + folinic acid + 5-FU), data were combined because information regarding dosing and schedule were unknown.

### Statistical analyses

The Kaplan-Meier method was used to determine OS, and a stratified log-rank test was used to assess statistical significance. In the case of patients who were lost to follow-up, survival data were censored at the last date at which they were known to be alive. The results presented herein are based on the updated cutoff date for OS analysis, which was 9 May 2013. Progression-free survival (PFS) was compared between the treatment arms using the Kaplan-Meier method, and differences were tested using a stratified log-rank test. For the OS and PFS analysis, the HR and 95 % CI calculation used the proportional hazard assumption. Differences in overall response rate (ORR) were assessed by *χ* ^2^ test.

## Results

### Baseline characteristics

In general, the baseline characteristics of patients treated to PD or AEs in the absence of PD were well balanced and similar to those of the intent-to-treat (ITT) population (Table 1). Although differences in baseline characteristics between the cohorts were not compared statistically, some minor imbalances were noted. Among patients treated with *nab*-P + Gem, those in the treatment-to-AEs cohort were older than those in the treatment-to-PD cohort or ITT population. Patients who received Gem alone in the treatment-to-AEs cohort had a greater metastatic burden compared with all other cohorts. Fewer patients in the treatment-to-AEs cohort underwent a previous Whipple procedure compared with those in the treatment-to-PD cohort and the ITT population. Among patients who were treated with Gem monotherapy, more patients in the treatment-to-AEs cohort had a biliary stent at baseline compared with those in the treatment-to-PD cohort and the ITT population.

**Table 1:** Baseline characteristics of patients treated to disease progression, adverse events in the absence of disease progression, and the intent-to-treat population

Patient characteristics	Patients treated to PD		Patients treated to AEs		ITT population[18]	
	*nab*-P + Gem (*n =* 224)	Gem (*n =* 233)	*nab*-P + Gem (*n =* 98)	Gem (*n =* 58)	*nab*-P + Gem (*n =* 431)	Gem (*n =* 430)
Age, median, years	61.0	63.0	66.5	63.5	62.0	63.0
≥ 65 years, %	39	42	55	48	41	44
Male, %, KPS, %	60	58	58	66	57	60
90 %-100 %	60	67	58	57	58	62
70 %-80 %	40	33	41	43	42	38
Current site(s) of metastasis, %						
Lung	33	40	38	52	35	43
Liver	90	84	84	79	85	84
No. of metastatic sites, %						
1	5	6	7	3	8	5
2	47	50	47	41	47	48
3	32	32	32	31	32	33
>3	15	13	14	24	14	15
Previous Whipple procedure, %	8	7	3	3	7	7
Biliary stent, %	21	15	17	28	19	16

*AE* adverse event, *Gem* gemcitabine, *ITT* intent to treat, *KPS* Karnofsky performance status, *nab-P nab*-paclitaxel, *PD* progressive disease

### Efficacy

#### Overall survival

**Fig. 1:**
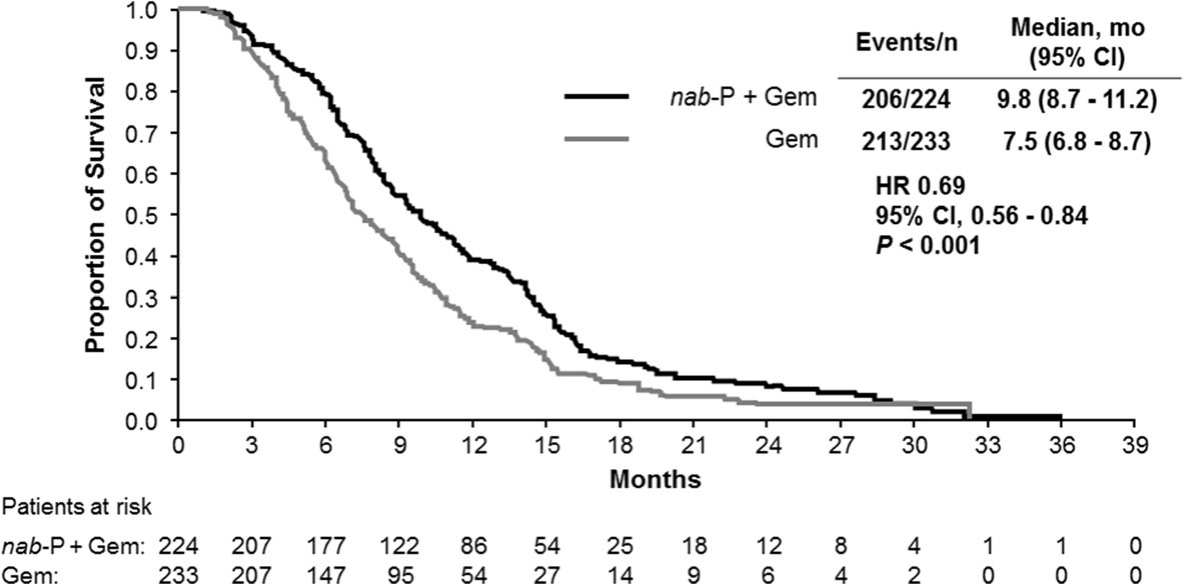
Overall survival in patients treated to disease progression. Gem, gemcitabine; HR, hazard ratio; *nab*-P, *nab*-paclitaxel

Overall survival in the treatment-to-PD cohort was significantly longer for patients who received *nab*-P + Gem compared with those who received Gem alone (median, 9.8 vs 7.5 months; HR, 0.69; *P* < 0.001; Fig. 1). Kaplan-Meier estimates of OS rate at 24 months following randomization were 8 % for *nab*-P + Gem compared with 4 % for Gem alone among patients in the treatment-to-PD cohort. The OS data in the treatment-to-PD cohort were based on 419 events (92 %), including 206 and 213 in the *nab*-P + Gem (92 %) and Gem-alone (91 %) arms, respectively.

Overall survival in the treatment to AEs cohort was numerically, but not significantly, longer for patients who received *nab*-P + Gem compared with those who received Gem alone (median, 7.7 vs 6.0 months; HR, 0.87; *P* = 0.466; Table 2 [based on 136 events; 87 %]). Kaplan-Meier estimates of OS rates at 24 months following randomization were 14 % for *nab*-P + Gem compared with 11 % for Gem alone among patients in the treatment-to-AEs cohort.

**Table 2:** Efficacy in patients treated to disease progression, adverse events in the absence of disease progression, and the intent-to-treat population

Efficacy variable	Patients treated to PD		Patients treated to AEs		ITT population [18, 19]	
	*nab*-P + Gem (*n =* 224)	Gem (*n =* 233)	*nab*-P + Gem (*n =* 98)	Gem (*n =* 58)	*nab*-P + Gem (*n =* 431)	Gem (*n =* 430)
Overall survival, median, months	9.8	7.5	7.7	6.0	8.7	6.6
Hazard ratio (95 % CI)	0.69 (0.56–0.84)		0.87 (0.60–1.27)		0.72 (0.62–0.83)	
*P* value	<0.001		0.466		<0.001	
Progression-free survival, median, months	6.0	3.8	5.5	5.0	5.5	3.7
Hazard ratio (95 % CI)	0.62 (0.50–0.79)		0.63 (0.40–1.01)		0.69 (0.58–0.82)	
*P* value	<0.001		0.053		<0.001	
Overall response rate, %	27	9	19	10	23	7
Complete response	<1	0	0	0	<1	0
Partial response	26	9	19	10	23	7
Stable disease	33	35	30	34	27	28
Response rate ratio (95 % CI)	3.12 (1.95–5.00)		1.87 (0.79–4.42)		3.19 (2.18–4.66)	
*P* value	<0.001		0.137		<0.001	
Disease control rate^a^	57	40	43^b^	42^c^	48	33
Disease control rate ratio (95 % CI)	1.42 (1.17–1.72)		1.03 (0.71–1.49)		1.46 (1.23–1.72)	
*P* value	<0.001		0.896		<0.001	

*AE* adverse event, *Gem* gemcitabine, *ITT* intent-to-treat, *nab-P nab*-paclitaxel, *PD* progressive disease

^a^Disease control rate includes patients who achieved a complete or partial response or stable disease for ≥ 16 weeks

^b^Based on 99 evaluable patients

^c^Based on 59 evaluable patients

#### Progression-free survival

**Fig. 2:**
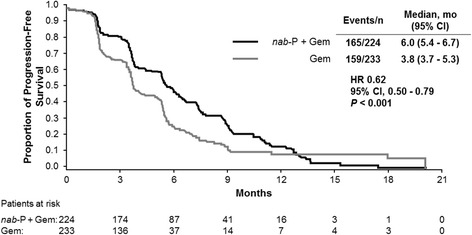
Progression-free survival in patients treated to disease progression. Gem, gemcitabine; HR, hazard ratio; *nab*-P, *nab*-paclitaxel

In patients treated to PD, PFS was significantly longer for patients treated with *nab*-P + Gem compared with those who received Gem alone (median, 6.0 vs 3.8 months; HR, 0.62; *P* < 0.001; Fig. 2). In patients treated to AEs, PFS was numerically longer for patients treated with *nab*-P + Gem compared with those who received Gem alone, although this difference did not reach statistical significance (median, 5.5 vs 5.0 months; HR, 0.63; *P* = 0.053).

#### Overall response rate

In the treatment-to-PD cohort, the independently assessed ORR was significantly higher for patients treated with *nab*-P + Gem vs those treated with Gem alone (27 % vs 9 %; response rate ratio [RRR], 3.12; *P* < 0.001; Table 2). One patient (<1 %) in the *nab*-P + Gem arm and 0 patients in the Gem-alone arm achieved a complete response (CR). The disease control rate (DCR; CR + partial response + stable disease for ≥ 16 weeks) was also significantly higher for patients in this cohort who were treated with *nab*-P + Gem compared with Gem alone (57 % vs 40 %; RRR, 1.42; *P* < 0.001).

The independently assessed ORR was numerically higher for patients in the treatment-to-AEs cohort who received *nab*-P + Gem vs Gem alone (19 % vs 10 %; RRR, 1.87; *P* = 0.137; Table 2). No patients in either treatment arm achieved a CR in this cohort. The DCR was comparable for patients in this cohort who received *nab*-P + Gem compared with Gem alone (43 % vs 42 %).

### Treatment exposure

The median treatment duration for patients in the treatment-to-PD cohort was 5.3 months (range, 0.16-21.9) for *nab*-P + Gem and 3.6 months (range, 0.13-21.5) for Gem alone (Table 3). For patients in the treatment-to-AEs cohort, the median treatment durations were 2.9 months (range, 0.13-20.7) and 2.3 months (range, 0.16-25.8), respectively (Table 3).

**Table 3:** Treatment exposure in patients treated to disease progression, adverse events in the absence of disease progression, and the overall treated population

Treatment exposure	Patients treated to PD		Patients treated to AEs		All treated patients	
	*nab*-P + Gem (*n =* 224)	Gem (*n =* 233)	*nab*-P + Gem (*n =* 98)	Gem (*n =* 58)	*nab*-P + Gem (*n =* 421)	Gem (*n =* 402)
Treatment duration, median, months	5.3	3.6	2.9	2.3	3.9	2.8
*nab*-P dose intensity, median, mg/m^2^/week	77.8	—	61.2	—	74.1	—
Gem dose intensity, median, mg/m^2^/week	626.3	695.9	523.4^a^	590.9	597.3	674.9
*nab*-P cumulative dose, median, mg/m^2^	1738	—	925	—	1425	—
Gem cumulative dose, median, mg/m^2^	13,000	10,000	8000^a^	7500	11,400	9000
*nab*-P doses given at 125 mg/m^2^, %	72	—	62	—	71	—
Gem doses given at 1000 mg/m^2^, %	65	75	53	81	63	79
Patients with ≥ 1 *nab*-P dose reduction, n (%)	103 (46)	—	37 (38)	—	172 (41)	—
Patients with ≥ 1 Gem dose reduction, n (%)	114 (51)	89 (38)	48 (49)	19 (33)	198 (47)	132 (33)
Patients with ≥ 1 *nab*-P dose delay, n (%)	165 (74)	—	72 (73)	—	300 (71)	—
Patients with ≥ 1 Gem dose delay, n (%)	158 (71)	145 (62)	72 (73)	36 (62)	295 (70)	230 (57)

*AE* adverse event, *Gem* gemcitabine, *ITT* intent-to-treat, *nab-P nab*-paclitaxel, *PD* progressive disease

^a^Based on 97 evaluable patients

Among patients treated to PD in the *nab*-P + Gem arm, 46 % had ≥ 1 *nab*-P dose reduction and 74 % had ≥ 1 *nab*-P dose delay (Table 3). Similarly, among patients treated to AEs in the *nab*-P + Gem arm, 38 % of patients had ≥ 1 *nab*-P dose reduction and 73 % had ≥ 1 *nab*-P dose delay (Table 3).

Among patients treated to PD in the *nab*-P + Gem arm, the percentage of *nab*-P doses delivered at 125 mg/m^2^ and Gem doses delivered at 1000 mg/m^2^ were 72 % and 65 %, respectively; 75 % of Gem doses were delivered at 1000 mg/m^2^ in the Gem-alone arm (Table 3). Among patients treated to AEs in the *nab*-P + Gem arm, the percentage of *nab*-P doses delivered at 125 mg/m^2^ and Gem doses delivered at 1000 mg/m^2^ were numerically lower (62 % and 53 %, respectively), and the percentage of Gem doses delivered at 1000 mg/m^2^ in the Gem-alone arm was numerically higher (81 %; Table 3). Cumulative doses are described in detail in Table 3.

### Reasons for treatment discontinuation

The AEs that most commonly led to treatment discontinuation are summarized in Table 4. Among patients in the treatment-to-AEs cohort who received *nab*-P + Gem, the most common AEs that led to treatment discontinuation were peripheral neuropathy and fatigue. Among patients in the treatment-to-AEs cohort who received Gem alone, the most common AE that lead to treatment discontinuation was thrombocytopenia.

**Table 4:** Most common adverse events that led to treatment discontinuation^a^

Adverse event, n (%)	*nab*-P + Gem (*n =* 98)	Gem (*n =* 58)
Peripheral neuropathy	17 (17)	0
Fatigue	11 (11)	2 (3)
Thrombocytopenia	7 (7)	7 (12)
Pneumonia	5 (5)	3 (5)
Pulmonary embolism	2 (2)	5 (9)
Vomiting	2 (2)	3 (5)
Cerebrovascular accident	0	3 (5)

*Gem* gemcitabine, *nab-P nab*-paclitaxel

^a^Most frequent was defined as those adverse events occurring in ≥ 5 % of the patients in either treatment arm

### Subsequent therapy use

Within the treatment-to-PD cohort, the use of subsequent therapy in the *nab*-P + Gem and Gem-alone arms was 52 % and 57 %, respectively, and these patients had numerically longer OS (median, 11.3 and 9.4 months, respectively; Table 5) than all patients in this cohort (median, 9.8 and 7.5 months, respectively; Fig. 1 and Table 2). In both arms of the treatment-to-PD cohort, 5-FU– or capecitabine-based regimens were the most commonly used subsequent therapies, with the majority of these patients having received a 5-FU–based regimen. Among patients who were treated to AEs, the majority (73 % and 74 % of those in the *nab*-P + Gem and Gem-alone arms, respectively) did not receive a subsequent therapy; therefore, OS was not reported for these patients.

**Table 5:** Subsequent therapy use in patients treated to disease progression

Subsequent therapies	Patients treated to PD	
	*nab*-P + Gem (*n =* 224)	Gem (*n =* 233)
Any subsequent therapy, n (%)	117 (52)	133 (57)
OS, median, months	11.3	9.4
HR (95 % CI)	0.75 (0.57-0.97)	
*P* value	0.027	
5-FU/capecitabine based, n (%)^a^	99 (85)	109 (82)
OS, median, months	11.6	9.2
HR (95 % CI)	0.71 (0.53–0.94)	
*P* value	0.017	
FOLFIRINOX (modified/unmodified), n (%)^a^	14 (12)	18 (14)
OS, median, months	15.3	7.6
HR (95 % CI)	0.45 (0.20–1.00)	
*P* value	0.044	
FOLFOX/OFF, n (%)^a^	27 (23)	37 (28)
OS, median, months	13.5	9.5
HR (95 % CI)	0.58 (0.34–0.98)	
*P* value	0.038	
Other, n (%)^a^	18 (15)	24 (18)
OS, median, months	10.0	10.4
HR (95 % CI)	1.00 (0.53–1.88)	
*P* value	>0.999	
No subsequent therapy, n (%)	107 (48)	100 (43)
OS, median, months	7.9	5.2
HR (95 % CI)	0.62 (0.46–0.82)	
*P* value	<0.001	

*5-FU* 5-fluorouracil, *Gem* gemcitabine, *FOLFIRINOX* folinic acid + 5-fluorouracil + irinotecan + oxaliplatin, *FOLFOX* folinic acid + 5-fluorouracil + oxaliplatin, *nab-P nab*-paclitaxel, *OFF* oxaliplatin + folinic acid + 5-fluorouracil, *OS* overall survival, *PD* progressive disease

^a^For specific examples of subsequent therapies, percentages are calculated using the number of patients who received a subsequent therapy as the denominator

### Safety

Incidences of grade ≥ 3 hematologic AEs in both cohorts were similar to those reported in the MPACT trial, although there were higher rates of anaemia among patients treated to AEs in both treatment arms compared with the treated population of the MPACT trial [18]. Among patients treated to PD, *nab*-P + Gem, compared with Gem alone, had slightly higher rates of neutropenia (39 % vs 31 %) and thrombocytopenia (13 % vs 9 %) but not anaemia (14 % each; Table 6). Among patients treated to AEs, *nab*-P + Gem, compared with Gem alone, had higher rates of neutropenia (40 % vs 27 %), but rates of anaemia (19 % vs 18 %) and thrombocytopenia (14 % each) were similar (Table 6). Febrile neutropenia occurred in 2 % of patients in each treatment arm of the treatment-to-PD cohort and in 4 % and 2 % of patients who received *nab*-P + Gem and Gem alone, respectively, in the treatment-to-AEs cohort (Table 6).

**Table 6:** Adverse events in patients treated to disease progression, adverse events in the absence of disease progression, and the overall treated population

Grade ≥ 3 AEs, %	Patients treated to PD		Patients treated to AEs		All treated patients [18]	
	*nab*-P + Gem	Gem	*nab*-P + Gem	Gem	*nab*-P + Gem	Gem
Haematologic^a^	*n =* 223	*n =* 233	*n =* 91	*n =* 56	*n =* 405	*n =* 388
Neutropenia	39	31	40	27	38	27
Leukopenia	29	18	41	21	31	16
Thrombocytopenia	13	9	14	14	13	9
Anaemia	14	14	19	18	13	12
Febrile neutropenia^b^	2	2	4	2	3	1
Nonhaematologic^c^	*n =* 224	*n =* 233	*n =* 98	*n =* 58	*n =* 421	*n =* 402
Fatigue	15	5	29	10	17	7
Peripheral neuropathy^d^	19	1	21	0	17	1
Diarrhoea	6	2	11	3	6	1

^a^Based on laboratory values (some missing values)

^b^Percentages were calculated using the n's reported for nonhaematologic AEs

^c^Based on investigator assessment of treatment-related events

^d^Grouped AE term

*AE* adverse event, *Gem* gemcitabine, *ITT* intent to treat, *nab-P nab*-paclitaxel, *PD* progressive disease

Incidences of grade ≥ 3 nonhematologic AEs in both cohorts were generally similar to those reported in the MPACT trial, although fatigue occurred more frequently among patients who received *nab*-P + Gem in the treatment-to-AEs cohort vs the treated population of the MPACT trial [18]. In both cohorts, rates of fatigue, peripheral neuropathy, and diarrhoea were higher for *nab*-P + Gem vs Gem alone (Table 6). The frequency of grade 2 peripheral neuropathy was similar for patients who received *nab*-P + Gem in the treatment-to-PD cohort, treatment-to-AEs cohort, and overall MPACT treated population (16 %, 13 %, and 15 %, respectively).

## Discussion

This subanalysis provides evidence that the *nab*-P + Gem combination is an active first-line treatment option with significant clinical efficacy for patients with metastatic pancreatic cancer because the majority of patients were treated until PD, which was associated with longer survival compared with the ITT population. Treatment until PD allowed better efficacy (as assessed by OS and disease control), likely due to the longer treatment duration and greater treatment exposure these patients received compared with those in the ITT population [18]. In addition, more than half of the patients who were treated to PD received a subsequent therapy, indicating that *nab*-P + Gem was also a feasible first-line option on which a treatment plan can be built. Conversely, ≈ 20 % of patients in the study discontinued treatment due to AEs in the absence of PD, which limited their treatment exposure and potential efficacy benefit.

A detailed examination revealed interesting differences in the relationships of reason for discontinuation, treatment exposure, and efficacy between the 2 treatment arms. Among patients who received *nab*-P + Gem, there was greater efficacy in terms of OS, PFS, and ORR between patients treated until PD vs the ITT population. Conversely, although there was a survival benefit in the Gem-alone arm between patients treated until PD vs the ITT population, the difference in PFS and ORR between the 2 cohorts was modest. The difference in treatment duration between patients treated until PD and the overall treated population was numerically longer for those who received *nab*-P + Gem compared with Gem alone (1.4 vs 0.8 months). The cumulative Gem dose in the PD cohort was 14 % and 11 % higher than that in the overall treated population for the *nab*-P + Gem and Gem-alone arms, respectively; however, the cumulative *nab*-P dose was 22 % higher for the PD cohort than the overall treated cohort for the combination arm (Table 3). These data raise the intriguing but speculative question of whether exposure to *nab*-P vs Gem imparts a greater relative treatment benefit.

Adverse events associated with chemotherapy are routinely managed by dose modification. A post hoc analysis of patients who underwent dose reductions or delays in the MPACT trial demonstrated that OS was significantly longer for those with vs without dose modifications [22]. Thus, mitigating AEs associated with *nab*-P + Gem through dose modification is not detrimental to treatment efficacy. The present analysis reveals that patients who were treated until PD had more dose reductions and delays compared with those in the ITT population, which suggests that effective treatment management may have allowed them to continue to receive and benefit from therapy.

The most common reasons for discontinuation due to AEs in the combination arm were peripheral neuropathy, fatigue, and thrombocytopenia. Rates of grade 3 peripheral neuropathy were relatively similar among patients treated to PD or AEs and the overall treated population, as were rates of grade 2 peripheral neuropathy. Management of peripheral neuropathy is accomplished by pausing treatment or reducing the treatment dose. Interestingly, in the MPACT trial, OS was significantly longer among patients who developed grade 3 peripheral neuropathy vs those who did not develop peripheral neuropathy [23]. Furthermore, dose modification was frequently used for patients who developed grade 3 peripheral neuropathy (≥1 dose delay [80 %] and/or reduction [41 %]). This approach for the management of peripheral neuropathy led to longer treatment duration, and ultimately, greater treatment exposure, which likely influenced survival outcomes. Collectively, these results underscore the importance of AE management through dose modification.

This subanalysis revealed that among patients who received *nab*-P + Gem, not only were the rates of grade ≥ 3 peripheral neuropathy similar in patients treated to PD vs AEs (19 % vs 21 %, respectively), but so were the rates of grade ≥ 3 neutropenia (39 % vs 40 %) and thrombocytopenia (13 % vs 14 %). Thus, grade ≥ 3 AEs were no less likely in patients who discontinued due to PD than in those who discontinued due to AEs, which may further underscore the importance of managing toxicity to maximize treatment duration.

Analysis of treatment effect by ORR is a direct measurement of antitumour activity and, unlike OS, which can be confounded by subsequent therapies, improvements in ORR can be directly attributed to the ongoing treatment [24]. Progression-free survival encompasses time to disease progression or death [24] and represents an important aspect of palliative treatment pancreatic cancer. Patients treated to AEs in the absence of PD still experienced treatment benefit, as evidenced by ORR and PFS analyses, which suggests that management of AEs before the need for discontinuation may have prolonged treatment and potentially increased survival. The shorter OS in patients treated to AEs is likely due to the shorter treatment duration and infrequent use of subsequent therapies among patients in this cohort. It also remains unanswered whether any of these patients could have resumed therapy outside of a protocol requirement, in which strict rules apply for AE management and treatment discontinuation.

Baseline characteristics were uninformative regarding identification of patients who may have developed treatment-limiting AEs during therapy. Compared with the other cohorts, fewer patients treated to AEs had a previous Whipple procedure, indicating a more advanced disease stage at diagnosis for these patients. Among patients who were treated until AEs, those in the *nab*-P + Gem arm were older while those in the Gem arm had a greater metastatic burden compared with those in the treatment-to-PD cohort as well as the ITT population. However, at baseline, the performance status of these patients was similar to that of the ITT population. At this point, whether these imbalances influenced survival outcomes is speculative. A future biomarker analysis may provide information regarding which patients are likely to benefit from treatment vs develop unacceptable AEs.

Historically, treatment beyond first line has been an option for a subset of patients with metastatic pancreatic cancer [25, 26]. Several randomized phase 2 clinical trials have explored second-line chemotherapy use in patients with metastatic pancreatic cancer [27–30]. Patients enrolled in these trials were all previously treated with Gem or Gem-based regimens, and efficacy results were modest. The current analysis, although not specifically designed to test this hypothesis, shows that, in the treatment-to-PD cohort, OS was numerically longer among patients who received a subsequent therapy compared with those who did not, regardless of treatment arm. The longest OS was achieved by those who received first-line *nab*-P + Gem followed by a subsequent therapy. A hypothetical explanation might be that first-line treatment with *nab*-P + Gem reduced tumor burden, which decreased cancer-related symptoms and ultimately allowed greater use of second-line therapies. These types of comparisons must be interpreted cautiously given the possibility of differences in patient characteristics, such as performance status, at the end of first-line treatment. However, > 50 % of patients who were treated to PD were able to receive subsequent therapy, which supports the suitability of *nab*-P + Gem as a first-line treatment for metastatic pancreatic cancer.

## Conclusions

The results presented herein emphasize that first-line treatment with *nab*-P + Gem can be optimized for maximum treatment benefit. As revealed by previous subanalyses of the MPACT trial, effective AE management (ie, by treatment delay or dose reduction) allows for longer treatment duration, which, in turn, increases treatment exposure [22, 23]. Therefore, physicians should pay close attention to and promptly address AEs, when possible, to allow treatment to PD. This MPACT subanalysis reveals that most patients treated to PD were able to achieve adequate treatment exposure while managing AEs, which translated to improved disease control and longer survival.

## Additional file

Additional file 1: List of Independent Ethics Committees and Institutional Review Boards for MPACT. (DOCX 39 kb)

## Abbreviations

5-FU: 5-fluorouracil
AE: Adverse event
CR: Complete response
DCR: Disease control rate
FOLFIRINOX: Folinic acid + 5-fluorouracil + irinotecan + oxaliplatin
FOLFOX: Folinic acid + 5-fluorouracil + oxaliplatin
Gem: Gemcitabine
HR: Hazard ratio
ITT: Intent to treat
KPS: Karnofsky performance status
MPACT: Metastatic Pancreatic Adenocarcinoma Clinical Trial
*nab*-P: *nab*-paclitaxel
OFF: Oxaliplatin + folinic acid + 5-fluorouracil
ORR: Overall response rate
OS: Overall survival
PD: Progressive disease
PFS: Progression-free survival
PRODIGE: Partenarait de Recherche en Oncologie Digestive
RRR: Response rate ratio
