# Supplementary material for: Efficacy and safety profile of nab-paclitaxel plus gemcitabine in patients with metastatic pancreatic cancer treated to disease progression: a subanalysis from a phase 3 trial (MPACT)
Source: BMC Cancer. 2016 Oct 21;16:817. doi: 10.1186/s12885-016-2798-8 (PMC5073820; doi:10.1186/s12885-016-2798-8)
Supplement: Additional file 1: — List of Independent Ethics Committees and Institutional Review Boards for MPACT. (DOCX 39 kb) [file 12885_2016_2798_MOESM1_ESM.docx]

**Additional File 1.** List of Independent Ethics Committees and Institutional Review Boards for MPACT

| Site No. | Name/Address of IEC/IRB |
| --- | --- |
| 0163 | Western Institutional Review Board 3535 Seventh Avenue Southwest Olympia, WA 98502-5010, USA |
| 0222 | Mercy IRB Cancer Resource Center 4300 West Memorial Road Oklahoma City, OK 73120, USA |
| 0273 | Piedmont Healthcare Institutional Review Board  1968 Peachtree Road, NW Atlanta, GA 30309, USA |
| 0433 | Western Institutional Review Board 3535 Seventh Avenue Southwest Olympia, WA 98502-5010, USA |
| 0448 | Comite d'ethique de la recherche clinique de l'Hotel-Dieu de Quebec 11 Cote du Palais  Quebec, Quebec G1R 2J6, Canada |
| 0452 | Western Institutional Review Board 3535 Seventh Avenue Southwest Olympia, WA 98502-5010, USA |
| 0616 | Western Institutional Review Board 3535 Seventh Avenue Southwest Olympia, WA 98502-5010, USA |
| 0618 | Western Institutional Review Board 3535 Seventh Avenue Southwest Olympia, WA 98502-5010, USA |
| 0624 | St. Mary Medical Center IRB 1201 Langhorne-Newton Road Langhorne, PA 19047, USA |
| 0662 | Western Institutional Review Board 3535 Seventh Avenue Southwest Olympia, WA 98502-5010, USA |
| 0702 | Allina Institutional Review Board 2925 Chicago Avenue South Mail Route 10105  Minneapolis, MN 55407, USA |

| 0719 | Mayo Clinic Institutional Review Board   1. First Street SW 2. Building, Room 4-60 Rochester, MA 55905, USA |
| --- | --- |
| 0736 | Ontario Cancer Research Ethics Board c/o Ontario Institute for Cancer Research  MaRS Centre, South Tower 101 College Street, Suite 800  Toronto, Ontario M5G 0A3, Canada |
| 0763 | Western Institutional Review Board 3535 Seventh Avenue Southwest Olympia, WA 98502-5010, USA |
| 0767 | Indiana University Institutional Review Board  Office of Research Administration 980 Indiana Avenue, Room 3315 Indianapolis, IN 46202-2915, USA |
| 0768 | Western Institutional Review Board 3535 Seventh Avenue Southwest Olympia, WA 98502-5010, USA |
| 0769 | Conjoint Health REB Office of Medical Bioethics  3330 Hospital Drive Northwest Heritage Medical Research Building, Room 93  Calgary, Alberta T2N 4N1, Canada |
| 0770 | Research Subjects' Protection Program University of Minnesota D-528 Mayo Memorial Building 420 Delaware Street Southeast Minneapolis, MN 55455, USA |
| 0771 | Western Institutional Review Board 3535 Seventh Avenue Southwest Olympia, WA 98502-5010, USA |
| 0772 | Western Institutional Review Board 3535 Seventh Avenue Southwest Olympia, WA 98502-5010, USA |
| 0773 | Johns Hopkins Medicine Institutional Review Board  1620 McElderry Street Reed Hall B-130  Baltimore, MD 21205-1911, USA |
| 0774 | Roswell Park Cancer Institute Institutional Review Board Elm and Carlton Streets Buffalo, NY 14263, USA |

| 0775 | Western Institutional Review Board 3535 Seventh Avenue Southwest Olympia, WA 98502-5010, USA |
| --- | --- |
| 0776 | Lahey Clinic, IRB 41 Mall Road  Burlington, MA 01805, USA |
| 0778 | Froedtert Hospital and Medical College of Wisconsin IRB 8701 Watertown Plank Road Milwaukee, WI 53226, USA |
| 0779 | Western Institutional Review Board 3535 Seventh Avenue Southwest Olympia, WA 98502-5010, USA |
| 0782 | Western Institutional Review Board 3535 Seventh Avenue Southwest Olympia, WA 98502-5010, USA |
| 0783 | Western Institutional Review Board 3535 Seventh Avenue Southwest Olympia, WA 98502-5010, USA |
| 0786 | Western Institutional Review Board 3535 Seventh Avenue Southwest Olympia, WA 98502-5010, USA |
| 0788 | Ontario Cancer Research Ethics Board c/o Ontario Institute for Cancer Research  MaRS Centre, South Tower 101 College Street, Suite 800  Toronto, Ontario M5G 0A3, Canada |
| 0790 | University of Illinois College of Medicine at Peoria Institutional Review Board  One Illini Drive, Box 1649 Peoria, IL 61656, USA |
| 0792 | Western Institutional Review Board 3535 Seventh Avenue Southwest Olympia, WA 98502-5010, USA |
| 0797 | Comite d'ethique de la recherche Hopital du Sacre-Coeur de Montreal 5400 ouest boul Gouin  Montreal, Quebec H4J 1C5, Canada |
| 0798 | Comite d'ethique de la recherche du CHUM  Edifice Cooper, Mezzanine 2, bureau M-207  3981 Boulevard St-Laurent  Montreal, Quebec H2W 1Y5, Canada |
| 0799 | U Mass Medical School Human Subjects Office  55 Lake Avenue North Worchester, MA 01655, USA |

| 0813 | Western Institutional Review Board 3535 Seventh Avenue Southwest Olympia, WA 98502-5010, USA |
| --- | --- |
| 0814 | Western Institutional Review Board 3535 Seventh Avenue Southwest Olympia, WA 98502-5010, USA |
| 0816 | Florida Hospital Institutional Review Board  212 E. Winter Park Street Orlando, FL 32804, USA |
| 0817 | Consortium IRB  Fred Hutchinson Cancer Research Center  1100 Fairview Avenue North Seattle, WA 98109, USA |
| 0818 | Mercy Health Springfield Communities Institutional Review Board  1235 East Cherokee Street Springfield, MO 65804, USA |
| 0819 | Western Institutional Review Board 3535 Seventh Avenue Southwest Olympia, WA 98502-5010, USA |
| 0821 | Western Institutional Review Board 3535 Seventh Avenue Southwest Olympia, WA 98502-5010, USA |
| 0823 | Western Institutional Review Board 3535 Seventh Avenue Southwest Olympia, WA 98502-5010, USA |
| 0980 | Western Institutional Review Board 3535 Seventh Avenue Southwest Olympia, WA 98502-5010, USA |
| 0981 | Western Institutional Review Board 3535 Seventh Avenue Southwest Olympia, WA 98502-5010, USA |
| 0982 | Western Institutional Review Board 3535 Seventh Avenue Southwest Olympia, WA 98502-5010, USA |
| 0983 | SUNY Upstate Medical University Institutional Review Board  for the Protection of Human Subjects 1109 Weiskotten Hall  750 East Adams Street Syracuse, NY 13210, USA |
| 0985 | Florida Hospital Institutional Review Board  212 E. Winter Park Street Orlando, FL 32804, USA |

| 1025 | Western Institutional Review Board 3535 Seventh Avenue Southwest Olympia, WA 98502-5010, USA |
| --- | --- |
| 1026 | University of Oklahoma Health Sciences Center IRB  1000 Stanton L. Young Boulevard, Suite 176  Oklahoma City, OK 73104, USA |
| 1031 | University of Utah Institutional Review Board  75 South 2000 East  Salt Lake City, UT 84112, USA |
| 1032 | University of British Columbia British Columbia Cancer Agency Research Ethics Board Fairmount Medical Building  6th Floor, #614  Vancouver, British Columbia V5Z 1H5, Canada |
| 1044 | Western Institutional Review Board 3535 Seventh Avenue Southwest Olympia, WA 98502-5010, USA |
| 1400 | Western Institutional Review Board 3535 Seventh Avenue Southwest Olympia, WA 98502-5010, USA |
| 1401 | Western Institutional Review Board 3535 Seventh Avenue Southwest Olympia, WA 98502-5010, USA |
| 1403 | Western Institutional Review Board 3535 Seventh Avenue Southwest Olympia, WA 98502-5010, USA |
| 1405 | Western Institutional Review Board 3535 Seventh Avenue Southwest Olympia, WA 98502-5010, USA |
| 1406 | Lakeland Regional Cancer Center Regulatory Committee  1324 Lakeland Hills Blvd. Lakeland, FL 33805, USA |
| 1408 | Western Institutional Review Board 3535 Seventh Avenue Southwest Olympia, WA 98502-5010, USA |
| 1409 | Western Institutional Review Board 3535 Seventh Avenue Southwest Olympia, WA 98502-5010, USA |
| 1410 | Western Institutional Review Board 3535 Seventh Avenue Southwest Olympia, WA 98502-5010, USA |
| 1411 | Western Institutional Review Board 3535 Seventh Avenue Southwest Olympia, WA 98502-5010, USA |

| 1412 | Western Institutional Review Board 3535 Seventh Avenue Southwest Olympia, WA 98502-5010, USA |
| --- | --- |
| 1442 | Western Institutional Review Board 3535 Seventh Avenue Southwest Olympia, WA 98502-5010, USA |
| 2000 | Phoebe Putney Memorial Hospital 417 West Third Avenue  Albany, GA 31701, USA |
| 2001 | Western Institutional Review Board 3535 Seventh Avenue Southwest Olympia, WA 98502-5010, USA |
| 2002 | Western Institutional Review Board 3535 Seventh Avenue Southwest Olympia, WA 98502-5010, USA |
| 2003 | Western Institutional Review Board 3535 Seventh Avenue Southwest Olympia, WA 98502-5010, USA |
| 2004 | University of Arkansas for Medical Sciences Institutional Review Board 4301 West Markham Street, Slot  #636  Little Rock, AR 72205, USA |
| 2005 | Sharp Health Care Institutional Review Board  8695 Spectrum Center Boulevard San Diego, CA 92123, USA |
| 2006 | Western Institutional Review Board 3535 Seventh Avenue Southwest Olympia, WA 98502-5010, USA |
| 2007 | Western Institutional Review Board 3535 Seventh Avenue Southwest Olympia, WA 98502-5010, USA |
| 2008 | Western Institutional Review Board 3535 Seventh Avenue Southwest Olympia, WA 98502-5010, USA |
| 5000 | US Oncology, Inc Institutional Review Board  10101 Woodloch Forest Drive The Woodlands, TX 77380, USA |
| 5001 | US Oncology, Inc Institutional Review Board  10101 Woodloch Forest Drive The Woodlands, TX 77380, USA |
| 5002 | US Oncology, Inc Institutional Review Board  10101 Woodloch Forest Drive The Woodlands, TX 77380, USA |

| 5003 | US Oncology, Inc Institutional Review Board  10101 Woodloch Forest Drive The Woodlands, TX 77380 , USA |
| --- | --- |
| 5004 | US Oncology, Inc Institutional Review Board  10101 Woodloch Forest Drive The Woodlands, TX 77380, USA |
| 5005 | US Oncology, Inc Institutional Review Board  10101 Woodloch Forest Drive The Woodlands, TX 77380, USA |
| 5006 | US Oncology, Inc Institutional Review Board  10101 Woodloch Forest Drive The Woodlands, TX 77380, USA |
| 5007 | US Oncology, Inc Institutional Review Board  10101 Woodloch Forest Drive The Woodlands, TX 77380, USA |
| 5008 | US Oncology, Inc Institutional Review Board  10101 Woodloch Forest Drive The Woodlands, TX 77380, USA |
| 5009 | US Oncology, Inc Institutional Review Board  10101 Woodloch Forest Drive The Woodlands, TX 77380, USA |
| 5011 | US Oncology, Inc Institutional Review Board  10101 Woodloch Forest Drive The Woodlands, TX 77380, USA |
| 5012 | US Oncology, Inc Institutional Review Board  10101 Woodloch Forest Drive The Woodlands, TX 77380, USA |
| 5013 | US Oncology, Inc Institutional Review Board  10101 Woodloch Forest Drive The Woodlands, TX 77380, USA |
| 5014 | US Oncology, Inc Institutional Review Board  10101 Woodloch Forest Drive The Woodlands, TX 77380, USA |
| 0752 | Cancer Institute NSW Clinical Research Ethics Committee  Level 9, 8 Central Ave, Australian Technology Park, Eveleigh, NSW 2015, Australia |

| 0755 | Bellberry HREC 229 Greenhill Road  Dulwich SA 5065 Australia |
| --- | --- |
| 0988 | Bellberry Human Research Ethics Committee  229 Greenhill Road  Dulwich SA Australia 5065 |
| 0996 | Cancer Institute NSW HREC Australian Technology Park Level 9, 8 Central Avenue  Eveleigh, NSW, 2015 Australia |
| 0997 | Melbourne Health HREC PO Melbourne Hospital, Parkville, VIC 3050 Australia |
| 0998 | Cancer Institute NSW HREC Australian Technology Park Level 9, 8 Central Avenue  Eveleigh, NSW, 2015 Australia |
| 0999 | Cancer Institute NSW HREC Australian Technology Park Level 9, 8 Central Avenue  Eveleigh, NSW, 2015 Australia |
| 1000 | Cancer Institute NSW HREC Australian Technology Park Level 9, 8 Central Avenue  Eveleigh, NSW, 2015 Australia |
| 1001 | Southern Health HREC Locked Bag 29  Clayton South, VIC 3169 Australia |
| 1002 | Bellberry Human Research Ethics Committees, 229 Greenhill Road, Dulwich, SA 5065 Australia |

| 1003 | Southern Adelaide Clinical Human Research Ethics Committee, Level 2, Room 2A221, Flinders Medical Centre, Bedford Park SA 5042 Australia |
| --- | --- |
| 1004 | Sir Charles Gairdner Group Human Research Ethics Committee  Level 2, A Block Hospital Ave,  Nedlands, WA, 6009 Australia |
| 1006 | Bendigo Health Care Group HREC, PO BOX 126, Bendigo, VIC, 30552  Australia |
| 1007 | Royal Brisbane and Women’s Hospital Human Research Ethics Committee  Butterfield Street Heston Q 4029 Australia |
| 1008 | Bellberry Human Research Ethics Committees, 229 Greenhill Road, Dulwich, SA 5065 Australia |
| 1009 | CNAH Human Research & Ethics Committee, 89 Strangways Terrace, North Adelaide SA 5006 Australia |
| 1010 | Bellberry Human Research Ethics Committees, 229 Greenhill Road, Dulwich, SA 5065 Australia |
| 1011 | Cancer Institute NSW HREC Australian Technology Park Level 9, 8 Central Avenue Eveleigh, NSW, 2015 Australia |
| 1012 | Human Ethics and Research Committee (Tasmania) Network, Office of Research Services, Private Bag 1, Hobart Tas 7001 Australia |
| 1013 | The Alfred Ethics Committee 2nd Floor, east Block  Alfred Hospital Commercial Road  Melbourne VIC 3004 Australia |

| 3010 | Ethik-Kommission des Landes Oberöstereich  Wagner-Jauregg Weg 15  Linz, Austria, 4020 |
| --- | --- |
| 3020 | Ethikkommission fur das Krankenhaus der Barmherzigen Schwestorn Linz  Seilerstatte 4  4020 Linz, Austria |
| 3030 | Ethikkommission des Landes Niederösterreich  Landhausplatz 1 Haus 15B  3109 St. Polten, Austria |
| 3040 | Ethikkommission der Medizinischen Universität Wien  Borschkegasse 8b/E06 1090 Wien, Austria |
| 3110 | AZ Groeninge - Ethisch Comite Campus Loofstraat  Loofstraat 43  8500 Kortrijk, Belgium |
| 3120 | Commissie Medische Ethiek – Toetsingscommissie  UZ KULeuven Herestraat 49  3000 Leuven, Belgium |
| 3130 | ULB Hôpital Erasme – Comité d’Ethique  Route de Lennik 808 Local 3W37  1070 Bruxelles, Belgium |
| 3140 | Imeldaziekenhuis v.z.w - Ethisch Comité  Imeldalaan 9  2820 Bonheiden, Belgium |

| 3150 | H-Hart Ziekenhuis Roeselare-Menen Vzw Medische Ethische Commissie Campus Menen  Rijselstraat 71  8930 Menen, Belgium |
| --- | --- |
| 3210 | CPP Ile-de-France VIII Hôpital Ambroise Paré  Laboratoire d'Anatomopathologie 9, avenue Charles de Gaulle  92100 Boulogne-Billancourt, France |
| 3220 | CPP Ile-de-France VIII Hôpital Ambroise Paré  Laboratoire d'Anatomopathologie 9, avenue Charles de Gaulle  92100 Boulogne-Billancourt, France |
| 3230 | CPP Ile-de-France VIII Hôpital Ambroise Paré  Laboratoire d'Anatomopathologie 9, avenue Charles de Gaulle  92100 Boulogne-Billancourt, France |
| 3310 | Ethikkommission der Med. Fakultat der LMU München  Petlenkoferstr. 8a  80336 München, Germany |
| 3320 | Ärztekammer Niedersachsen Ethikkommission  Berliner Allee 20  30175 Hannover, Germany |
| 3330 | Ethikkommission der Med. Fakultat der LMU München  Petlenkoferstr. 8a  80336 München, Germany |
| 3340 | Ethikkommission der Ärztekammer Nordrhein  Tersteegenstr. 9  40474 Düsseldorf, Germany |

| 3350 | Ethikkommission der Bayerischen Landesarztekammer  Muehlbaurstr. 16  81677 München, Germany |
| --- | --- |
| 3360 | Ethikkommission der Landesarztekammer Baden- Wuerttemberg  Jahnstrasse 40  70597 Stuttgart, Germany |
| 3405 | Comitato Etico Scientifico dell'Azienda Ospede Niguarda Ca Granda di Milano  Plazza Ospedale Maggiore 3 20162 Milano, Italy |
| 3410 | Comitato di Bioetica Fondazione IRCCS Policlinico S. Matteo di Pavia 27100 Pavia, Italy |
| 3415 | Comitato Etico  IRCCS Istituto Clinico Humanitas Via A. Manzoni 56  20089 Rozzano, Italy |
| 3420 | Comitato Etico IRCCS  Istituti Fisioterapici Ospitalieri Via Elio Chianesi, 53  00128 Roma, Italy |
| 3425 | Comitato Etico IRCCS  FondazioneCentro San Raffaele del MonteTabor  Via Olgettina, 60  20132 Milano, Italy |
| 3430 | Comitato Etico per la Sperimentazione dell' Azienda Ospedaliera Universitaria Integrata di Verona  c/o Ospedale Civile Maggiore P.le A.Stefani, 1  37134 Verona, Italy |

| 3435 | Comitato Etico IRCCS Istituto Oncologico Veneto Via Gattamelata 64  35128 Padova, Italy |
| --- | --- |
| 3440 | Comitato Etico dell'IRCCS  Istituto Nazionale per la Ricerca sul Cancro  Largo Rosanna Benzi, 10 16132 Genova, Italy |
| 3445 | Comitato Etico Dell'IRCCS Ospedale Oncologico di Bari Viale Orazio Flacco 65 70124 Bari, Italy |
| 3450 | Comitato per la Sperimentazione Clinica dei Medicinali Azienda Ospedaliera Universitaria Pisana  Via Roma 67  56126 Pisa, Italy |
| 3455 | Comitato Etico  IRCCS Casa Sollievo della Sofferenza  Via S. Leonardo  71013 San Giovanni Rotondo, Italy |
| 3460 | Comitato Etico IRCCS Istituto Europeo di Oncologia Via Ripamonti 435  20141 Milano, Italy |
| 3465 | Comitato Etico  Provinciale di Reggio Emilia Viale Risorgimento 57 42100 Reggio Emilia, Italy |
| 3470 | Comitato Etico  Ente Ospedaliero Ospedali Galliera Mura delle Cappuccine 14  16128 Genova, Italy |

| 3510 | Agencia de Ensayos Clinicos Hospital Universitari Vall d'Hebron Edifici Institut de Recerca, 3a Planta Pg. Vall d'Herbron, 119-129  08035 Barcelona, Spain |
| --- | --- |
| 3520 | Secretaria C.E.I.C Grupo HM  Hospital Universitario Madrid Montepríncipe  Avd de Monteprincipe, 25  28660 Madrid (Boadilla del Monte) , Spain |
| 3530 | Comité Autonomico de Ensayos Clinicos (CAEC)  Consejeria de Salud  Avd. de la Innovation, s/n Edificio Arena 1  41020 Sevilla, Spain |
| 3560 | Comité Autonomico de Ensayos Clinicos (CAEC)  Consejeria de Salud  Avd. de la Innovation, s/n Edificio Arena 1  41020 Sevilla, Spain |
| 3540 | Hospital Clinico San Carlos Fundacion para la Investigation  Servicio Farmacologia Clinica, 1a Planta - Ala Norte - Puerta G  C/. Profesor Martin Lagos, s/n 2804 Madrid, Spain |
| 3550 | Secretaria Comité Etico de Investigation Clinica  Hospital 12 de Octubre  Edificio Materno-Infantil, sotano 21 Avda. de Cordoba s/n  28041 Madrid, Spain |

| 3570 | Comité Etico de Investigation Clinica Hospital Clinic i Provincial  Agencia de Ensayos Clinicos- Servicio de Farmacia  C/. Villarroel, 170 - Sotano, Esc. 6B 08036 Barcelona, Spain |
| --- | --- |
| 3580 | Comité Ético de Investigación Clínica Hospital Ramón y Cajal  Carretera de Colmenar Viejo, km. 9, 100  28034 Madrid, Spain |
| 0351 | Ethics Committee within Central Clinical Hospital #2 n.a. Semashko under OJSC Russian Railways  2 Budaiskaya Ul., 129128, Moscow Russia |
| 0353 | Ethics Committee within St. Petersburg Municipal Oncology Center  56, Veteranov Pr., St. Petersburg, 198255, Russia |
| 0357 | Ethics Committee within Russian Oncological Research Center n.a.  N.N. Blokhin under RAMS  24 Kashirskoye Shosse, Moscow, 115478 Russia |
| 1034 | Ethics Committee within Russian Research Center for Radiology and Surgical Technologies under Federal Agency for High-Tech Medical Care  70 Leningradskaya Ul., Pesochny, St. Petersburg, 197758, Russia |
| 1035 | Local Independent Ethics Committee within Leningrad Regional Clinical Hospital  45-49 Lunacharskogo Pr., St. Petersburg, 194291, Russia |
| 1037 | Ethics Committee within Clinical Hospital #122 named after L.G. Sokolov under the Federal Medical- Biological Agency  4 Kultury pr., St. Petersburg, 194291, Russia |

| 1039 | Ethics Committee within Russian Research Center of Surgery n.a. B.V. Petrovskiy under the Russian Academy of Medical Sciences  2 Abrikosovsky Per., Moscow, 119992, Russia |
| --- | --- |
| 1041 | Ethics Committee within Moscow Municipal Oncological Hospital #62 under Moscow Department of Healthcare  Stepanovskoye, Krasnogorskiy District, Moscow Region, 143423, Russia |
| 1042 | Ethics Committee within  State Educational Institution of Higher Professional Education St. Petersburg State Medical Academy n.a. I.I. Mechnikov under the Federal Agency for  Healthcare and Social Development  41, Kirochnaya Ul, St. Petersburg, 191015, Russia |
| 1413 | Expert Board for Medical Care within Russian Research Center of Radiology under the Federal Agency for Hi-Tech Medical Care  86 Profsoyuznaya str., Moscow, 117997, Russia |
| 1414 | Ethics Committee within Central Clinical Hospital and Polyclinic under the President’s Business Administration Directorate of the Russian Federation  15 Marshala Timoshenko str., Moscow, 121359, Russia |
| 1416 | Ethics Committee within Municipal Clinical Hospital #57 under Moscow Department for Healthcare  32, 11-th Parkovaya str., Moscow, 105077, Russia |
| 1417 | Ethics Committee within Medical Radiological Research Center under RAMS  4 Koroleva Ul., Obninsk, Kaluga Region, 249036 Russia |

| 1423 | Local Ethics Committee within Pyatigorsk Oncological Center  31 Kalinina Pr., Pyatigorsk, Stavropol Territory, 357502, Russia |
| --- | --- |
| 1424 | Ethics Committee within  Ivanovo Regional Oncology Center  5 Lubimova Ul., Ivanovo, 153013, Russia |
| 1425 | Ethics Committee within "Republican Clinical Oncology Center under the Ministry of Healthcare of the Republic of Tatarstan"  29 Sibirskiy Trakt, Kazan, 420029, Republic of Tatarstan, Russia |
| 1426 | Ethics Committee within Tula Regional Oncology Center”  1B Yablochkova Ul., Tula, 300053, Russia |
| 1427 | Ethics Committee within Omsk Regional Budget Medical Institution: "Clinical Oncological Center",  Block 1, 9 Zavertiayeva Ul., Omsk,  644013, Russia |
| 1428 | There is no local ethics committee at Regional Clinical Oncology Hospital (State Medical Institution of Yaroslavl) |
| 1429 | Ethics Committee Chelyabinsk Regional Clinical Oncology Center  42 Blyukhera Ul., Chelyabinsk, 454087, Russia |
| 1430 | Ethics Committee within Republican Clinical Oncological Center  73/1, Oktiabria pr., Ufa, 450054, Russia |
| 1431 | Ethics Committee Altai Regional Oncology Center  77 Nikitina Ul., Barnaul, 656049, Russia |

| 1432 | Ethics Committee for Clinical Studies within Orenburg Regional Clinical Oncology Center  11 Gagarina Pr., Orenburg, 460021, Russia |
| --- | --- |
| 1433 | Ethics Committee within Regional Oncological Center # 2  4, Hertzen St., Magnitogorsk, 455001, Russia |
| 1418 | Ethics Committee within Public Institution: Dnipropetrovsk City Multispecialty Clinical Hospital #4 under Dnipropetrovsk Regional Council  31 Blyzhnia Vul., Dniprorpetrovsk, 49102, Ukraine |
| 1419 | Local Ethics Committee within Lviv State Regional Treatment and Diagnostics Oncology Center  2a Hasheka Vul., Lviv, 79031, Ukraine |
| 1420 | Ethics Committee within the Public Healthcare Institution: Kharkiv Regional Clinical Oncology Center  4 Lisoparkova Vul., 61070 Kharkiv, Ukraine |
| 1421 | Bioethics Committee within City Clinical Hospital #3  9 Metalurhiv Pr., 69032, Zaporizhia, Ukraine |
| 1422 | Bioethics Committee within Kharkiv Regional Clinical Oncology Center  4 Lisoparkova Vul., Kharkiv, 61070, Ukraine |
| 1434 | Ethics Committee within  Volyn Regional Oncology Center  1 Timiriazieva Vul., Lutsk, 43018, Ukraine |

| 1435 | Ethics Committee within the Public Clinical Treatment and Prophylaxis Institution: Donetsk Regional Antitumor Center  2a Polotska Vul., 83092, Donetsk, Ukraine |
| --- | --- |
| 1436 | Ethics Committee within the National Institute of Cancer  33/43 Lomonosova Vul., 0.3022, Kiev, Ukraine |
| 1437 | Ethics Committee within Zhytomyr O.F. Herbachevskyi Regional Clinical Hospital  3 Chervonoho Khresta Vul., Zhytomyr, 10008, Ukraine |
| 1438 | Ethics Committee within Kherson Regional Oncology Center  26-Б Kindiiske Shose, Kherson, 73000, Ukraine |
| 1439 | Ethics Committee Kirovohrad Regional Oncology Center  1 Yaltynska Vul., 25031, Kirovohrad, Ukraine |
| 1440 | Ethics Committee within  Odesa Regional Clinical Hospital  26 Akademika Zabolotnoho Vul, 65117, Odesa, Ukraine |
| 1441 | Ethics Committee within  Kyiv City Clinical Hospital #10  59A 40-richia Zhovtnia Pr., 3039, Kiev, Ukraine |
